# Incidence rates and trends of childhood urinary tract infections and antibiotic prescribing: registry-based study in general practices (2000 to 2020)

**DOI:** 10.1186/s12875-022-01784-x

**Published:** 2022-07-20

**Authors:** Hanne A. Boon, Thomas Struyf, Jonas Crèvecoeur, Nicolas Delvaux, Gijs Van Pottelbergh, Bert Vaes, Ann Van den Bruel, Jan Y. Verbakel

**Affiliations:** 1 Academic Centre for General Practice, Department of Public Health and Primary Care, KU Leuven, Leuven, Belgium; 2 EPI-Centre, Department of Public Health and Primary Care, KU Leuven, Kapucijnenvoer 7, 3000 Leuven, Belgium; 3 L-BioStat, Department of Public Health and Primary Care, KU Leuven, Leuven, Belgium; 4 Nuffield Department of Primary Care Health Sciences, University of Oxford, Oxford, UK

**Keywords:** Retrospective studies, Incidence, General practice, General practitioners, Urinary tract infections, Urinalysis, Anti-bacterial agents

## Abstract

**Background:**

To improve the management of childhood urinary tract infections, it is essential to understand the incidence rates, testing and treatment strategy.

**Methods:**

A retrospective study using data from 45 to 104 general practices (2000 to 2020) in Flanders (Belgium). We calculated the incidence rates (per 1000 person-years) of cystitis, pyelonephritis, and lab-based urine tests per age (< 2, 2-4, 5-9 and 10-18 years)) and gender in children and performed an autoregressive time-series analysis and seasonality analysis. In children with UTI, we calculated the number of lab-based urine tests and antibiotic prescriptions per person-year and performed an autoregressive time-series analysis.

**Results:**

There was a statistically significant increase in the number of UTI episodes from 2000 to 2020 in each age group (*p* < 0.05), except in boys 2-4 years. Overall, the change in incidence rate was low. In 2020, the incidence rates of cystitis were highest in girls 2-4 years old (40.3 /1000 person-years 95%CI 34.5-46.7) and lowest in boys 10-18 (2.6 /1000 person-years 95%CI 1.8-3.6) The incidence rates of pyelonephritis were highest in girls 2-4 years (5.5, 95%CI 3.5-8.1 /1000 person-years) and children < 2 years of age (boys: 5.4, 95%CI 3.1-8.8 and girls: 4.9, 95%CI 2.7-8.8 /1000 person-years). In children 2-10 years, there was an increase in number of lab-based urine tests per cystitis episode per year and a decrease in total number of electronic antibiotic prescriptions per cystitis episode per year, from 2000 to 2020. In children with cystitis < 10 years in 2020, 51% (95%CI 47-56%) received an electronic antibiotic prescription, of which the majority were broad-spectrum agents.

**Conclusions:**

Over the last 21 years, there was a slight increase in the number of UTI episodes diagnosed in children in Flemish general practices, although the overall change was low. More targeted antibiotic therapy for cystitis in accordance with clinical guidelines is necessary to reduce the use of broad-spectrum agents in children below 10 years.

**Supplementary Information:**

The online version contains supplementary material available at 10.1186/s12875-022-01784-x.

## Introduction

Urinary tract infections (UTIs) can be potentially serious in children, resulting in hospitalization or long-term complications such as renal scarring [1]. Therefore, active assessment of the risk of UTI and prompt antibiotic treatment is recommended in several guidelines [2, 3]. Early diagnosis is challenging in primary care, because clinical signs and symptoms of UTI are nonspecific in children [4]. The urine dipstick test can help to assess the risk of UTI at the point-of-care. However, to confirm the presence of UTI in children, laboratory-based urine culture is generally required [2, 3].

There is limited data on the primary care-based diagnostic approach for UTI in children. Available numbers on the incidence rates of UTIs in children are based on either hospital data [5–9], or datasets of more than 20 years old [10–13]. More recent information on the number and time-trends of UTIs and urine testing is necessary to understand how often UTIs are diagnosed in children in general practice and whether the number of UTI diagnoses in primary care has changed.

Due to clinical diagnostic uncertainty, a long turnaround time of urine culture and a high contamination rate; early targeted antibiotic therapy for UTI is challenging. For cystitis in children, nitrofurantoin is recommended as first choice treatment in Belgian primary care guidelines since 2003, whereas for other infections, broad-spectrum antibiotics such as amoxicillin or amoxicillin clavulanic acid are commonly used in children [14]. When pyelonephritis is suspected, children should be referred to secondary care in Belgium [14]. Previous studies reported that clinical guidelines are often not followed for UTI in children [10]. However, antibiotic therapy should be targeted as much as possible, because infections with resistant pathogens are associated with significant morbidity [15]. Insight into the prescribing patterns is important to understand whether management of UTIs in Flemish general practice could be improved.

Therefore, the aim of this study was three-fold: to investigate the incidence rates and time-trends of UTIs and laboratory urine testing in children per age and gender and to investigate the number of laboratory urine tests and antibiotic prescriptions in children with UTI per age from 2000 to 2020 in primary care.

## Methods

### Study design and setting

This was a retrospective, registry-based study using data provided by the Intego project, which is a registration network as part of the Academic Centre of General Practice of the KU Leuven [16]. This study has been reported according to the EQUATOR guideline of Reporting of studies Conducted using Observational Routinely-collected health Data (RECORD) [17].

Intego collects health data in 45-104 general practices that are well spread across Flanders (Belgium). Information is routinely collected from the electronic health record and includes coded diagnoses, any laboratory tests such as urine culture and blood tests and drug prescriptions. Information from consultations in regular GP practice as well as during home visits are included. For this study, we used data per year starting from the first year of registration (2000) until the last full year available (2020). Starting from 2017 to 2018, more general practices could enter the registration network and additionally, the software for data registration changed from Medidoc to Careconnect. Therefore, the number of patients increased in 2018 and decreased in 2020 (Additional file 1).

### Population

To investigate UTI diagnosis and treatment in primary care, we selected two populations:

1. All children consulting a general practitioner (GP), to investigate the incidence rates of UTIs and laboratory urine testing. In Belgium, a large proportion of children are registered in a general practice. Per year, we included all children up to 18 years of age that visited the general practice at least once in that year, i.e. the yearly contact group. Children who did not visit the GP over the course of one full year and children turning older than 18 years, were not included for that corresponding year.
2. Children with UTI, to investigate the type and number of antibiotic prescriptions and number of laboratory urine tests. Per year, we included children up to 18 years visiting the general practice at least one, with a diagnosis of cystitis or pyelonephritis.

### Outcome measures

GPs register diagnoses in the patient file, using the International Classification of Primary Care codes (ICPC-2). In this study, UTI was defined as either cystitis (code U71) or pyelonephritis (ICPC-2 code U70), as registered by the GP. When two such codes were registered for the same patient, these were considered as two separate episodes if they were at least 8 weeks apart, whereas entries less than 8 weeks apart were considered as belonging to the same illness episode assuming an 8 weeks’ time-to-recovery per patient [18].

A urine test was defined as one or more of the following lab-based tests: urine dipstick test, urine microscopy, urine culture, antibiotic susceptibility pattern, taking into account 2 weeks’ time overlap as correction for multiple testing during the same illness episode. In children with UTI, tests had to be registered within 2 weeks before or after UTI diagnosis. Only lab-based tests were included, tests performed at the general practice were not included as these were not available in the database. To select eligible urine tests, we used both coded labels and free text labels.

Antibiotic therapy for UTI was defined as an electronic antibiotic prescription, based on any of the Anatomical Therapeutic Chemical Classification (ATC) codes for systematic use (J01), registered within 2 weeks before or after UTI diagnosis (ICPC-2 code U70, U71). Compound prescriptions, i.e. manual non-electronic prescriptions, were not available and therefore not included.

### Statistical analyses

The statistical analyses were performed using SAS Statistical Software version 9.4 (SAS Institute Inc., Cary, NC) and R Statistical Software version 4.1.1 (R Foundation, Austria).

The incidence rates per 1000 person-years in children visiting the general practice and 95% confidence intervals (95%CI) of cystitis, pyelonephritis, and urine testing were calculated per age group (< 2 years, 2-5 years, 5-9 years and 10-18 years) and gender; by dividing the number of new infections or tests over the total person-time at risk (package ‘epiR’ in R). An overview of the incidence rates was plotted in R using package ‘ggplot2’. Differences in incidence rates between subgroups were evaluated descriptively, by assessing the 95% CI.

Time-trends of cystitis and pyelonephritis were calculated using an autoregressive moving average time-series analysis per age group and gender from 2000 to 2020, taking into account data correlation over subsequent years [19]. Stable and moving seasonality of cystitis and pyelonephritis (2000-2020) was calculated using the ‘PROC X12 procedure’ in SAS, when at least one episode per month was available [20].

The number of laboratory urine tests per episode per year were calculated per age group; by dividing the number of tests over the total person-time at risk (package ‘epiR’ in R).

The number of antibiotic prescriptions per episode per year was calculated based on the absolute number of prescriptions per antibiotic type over the total number of cystitis or pyelonephritis episodes. The number and type of antibiotic prescriptions were plotted per age group. We grouped the type of antibiotic prescriptions into categories, as relevant for clinical care. An autoregressive moving average time-series analysis (2000-2020) per age group was performed for the total number of prescriptions and subtypes of antibiotic prescriptions when a time-trend was suspected based on visual inspection of the plot.

## Results

### Population

The yearly number of participating general practices and the difference in the yearly contact group (children visiting the general practice) and the practice population (including children not visiting the general practice) is shown in Additional file 1. The average number of GPs in 2019 was 4.86. The yearly contact group of children ≤18 years of age increased from 16,330 (2000) to 56,651 (2020).

From 2000 to 2020, there were 6970 children with cystitis and 707 children with pyelonephritis episodes in total. Of those, a minority of children had repeated UTI episodes: 5.8% (*n* = 404/6970) of children had two or more cystitis episodes and 4.0% (*n* = 28/707) of children had two or more pyelonephritis episodes.

### Incidence rates, seasonality and time-trends of UTI in children (2000-2020)

#### Incidence rates UTI

**Fig. 1:**
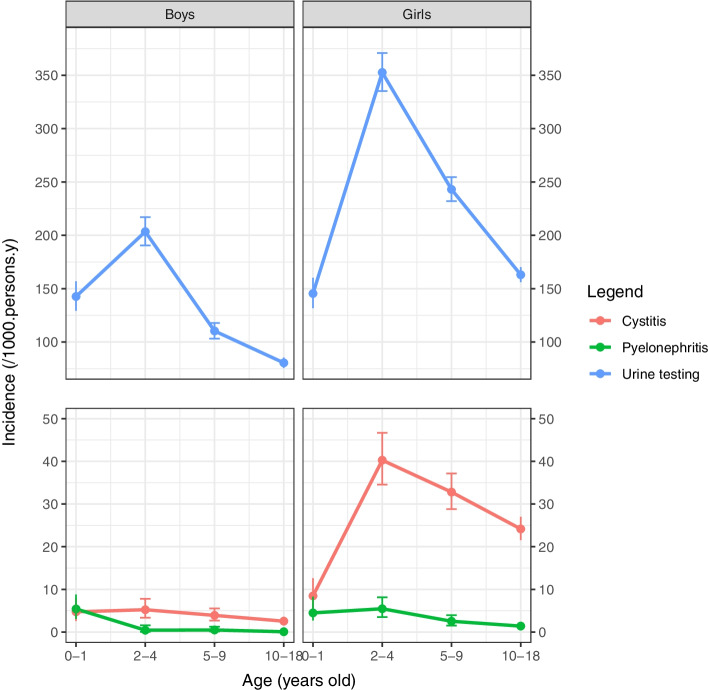
Incidence rates of cystitis, pyelonephritis, and urine testing in children per age and gender (2020). Incidence rates (per 1000 person-years) of cystitis (red), pyelonephritis (green) and urine testing (blue) in children < 18 years per age and gender (2020); Urine testing was defined as any of the following laboratory-based tests: urine dipstick, urine microscopy, urine culture or antibiotic susceptibility pattern

In 2020, urine testing rate was highest in children between 2 and 4 years old (Fig. 1, Additional file 2). In 2020, the incidence rates of cystitis were highest in 2-4 years old girls (40.3 per 1000 person-years 95%CI 35.54-46.7) and 5-9 years old girls (32.8 per 1000 person-years 95%CI 28.8-37.2) (Fig. 1, Additional file 2). The incidence rates of cystitis were lowest in boys ≥10 years of age (2.6 per 1000 person-years 95%CI 1.8-3.6). At all ages, the incidence rates of cystitis were significantly higher in girls than in boys (3 to 11 times higher). For pyelonephritis, the incidence rates were highest in young children < 2 years old, and girls 2-4 years (Additional file 2).

#### Seasonality

Independent of age and gender, cystitis rates followed a seasonal pattern (*p* < 0.001), with a slight decrease in incidence rate during June to August (Additional file 3). There was no moving seasonality for cystitis (*p* = 0.2140), meaning the stable seasonality pattern did not change significantly over the years. For pyelonephritis, stable and moving seasonality could not be assessed, because the number of episodes per month was too low.

#### Time-trends UTI

**Fig. 2:**
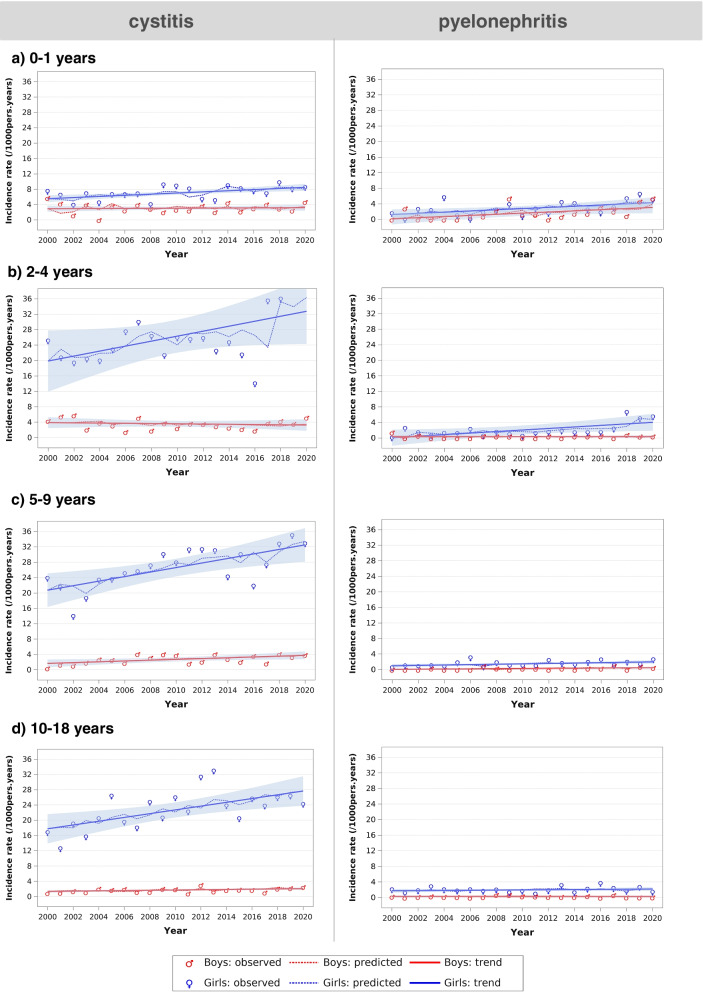
Trends of incidence rates of cystitis (left) and pyelonephritis (right) in girls and boys (2000-2020). Incidence rates (per 1000 person-years) of cystitis and pyelonephritis per age and gender from 2000 to 2020. (See Table 1 for the results of the autoregressive moving average time series analysis)

From 2000 to 2020, there was a statistically significant increase of cystitis episodes for all children ≥5 years old and girls < 2 years old (Table 1, Fig. 2). The highest increase in cystitis episodes was observed for girls 5-9 years, where it changed from 19.4 per 1000 person-years (95%CI 16.2-23.0) (2000-2002) to 33.4 per 1000 person-years (95%CI 31.0-35.9) (2018-2020).

**Table 1:** Autoregressive moving average time series analysis of trends of cystitis and pyelonephritis in children (2000-2020)

Infection	Age group (Years)	Gender	Annual change in incidence rate^**1**^ (/1000 person-years (95%CI))	***P***-value
Cystitis	0-1	Girls	**+ 0.1481 (0.0721 to 0.2241)**	**0.0010**
		Boys	−0.0135 (− 0.0537 to 0.0807)	0.6873
	2-4	Girls	+ 0.6453 (−0.0319 to 1.3225)	0.0690
		Boys	−0.0312 (− 0.1509 to 0.0886)	0.6028
	5-9	Girls	**+ 0.5901 (0.2321 to 0.9481)**	**0.0037**
		Boys	**+ 0.1049 (0.0214 to 0.1884)**	**0.0204**
	10-18	Girls	**+ 0.4956 (0.1755 to 0.8157)**	**0.0058**
		Boys	**+ 0.0360 (0.0092 to 0.0628)**	**0.0139**
Pyelonephritis	0-1	Girls	**+ 0.1521 (0.0395 to 0.2648)**	**0.0136**
		Boys	**+ 0.1466 (0.0238 to 0.2695)**	**0.0264**
	2-4	Girls	**+ 0.1977 (0.0251 to 0.3704)**	**0.0322**
		Boys	−0.0000 (−0.0284 to 0.0284)	0.9987
	5-9	Girls	**+ 0.0496 (0.0111 to 0.0881)**	**0.0177**
		Boys	**+ 0.0222 (0.0058 to 0.0387)**	**0.0135**
	10-18	Girls	+ 0.0217 (−0.0190 to 0.0624)	0.2934
		Boys	−0.0007 (− 0.0197 to 0.0184)	0.9419

*95%CI* 95% confidence intervals

^1^Mean annual change in incidence rate as estimated by the model; Statistically significant changes are indicated in **bold** (*p* < 0.05)

For pyelonephritis, there was a statistically significant increase in number of episodes for children < 2 years old, 5-9 years old and girls 2-4 years old from 2000 to 2020 (Table 1). The highest increase in pyelonephritis episodes was found for boys < 2 years, where it changed from 0.9 per 1000 person-years (95%CI 0.1-3.3) (2000-2003) to 3.4 per 1000 person-years (95%CI 2.5-5.0) (2017-2020).

### Incidence rates and time-trends of lab-based urine tests and antibiotic prescriptions in children with UTI (2000-2020)

#### Urine testing rates

In children with cystitis < 10 years, the number of laboratory-based urine tests per episode increased from 2000 to 2020 (*p* < 0.05, Additional files 4 and 5). In 2020, the number of lab-based tests per cystitis episode was 0.62 (95%CI 0.56 to 0.70 per person-years) in children < 10 years; whereas in 2000, the number of lab-based tests per cystitis episode was 0.11 (95%CI 0.06-0.21 per person-years) in children < 10 years. In children above 10 years of age, the number of lab-based tests per episode did not change significantly (*p* = 0.085), and remained at 0.53 per person-years (95%CI 0.46-0.62) in 2020.

#### Antibiotic prescribing rates

In children with cystitis above 2 years, there was a decrease in total number of electronic antibiotic prescriptions per episode per year from 2000 to 2020 (Additional file 5). In 2020, the number of prescriptions per cystitis episode was 0.54 (95%CI 0.37-0.69), 0.50 (95%CI 0.43-0.57), 0.52 (95%CI 0.46-0.58), and 0.85 (95%CI 0.81-0.89%) for children < 2 years, 2-5 years, 5-9 years and 10-18 years respectively. In children that did not receive antibiotic therapy, 59% (95%CI 54-65%) had a lab-based urine test performed.

**Fig. 3:**
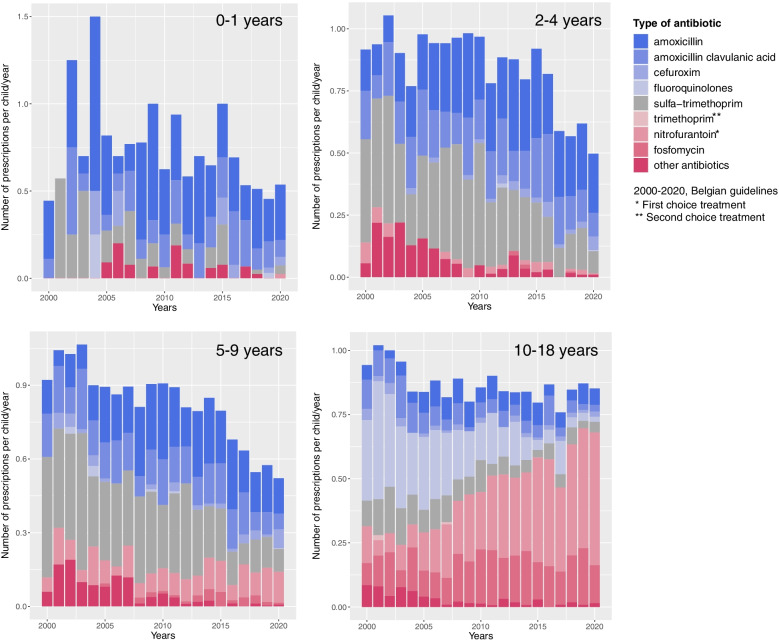
Number and type of antibiotic prescriptions per person-year in children with cystitis (2000 to 2020). The number of antibiotic prescriptions (per person-year) in children with cystitis per age (0-1 years old, 2-4 years old, 5-9 years old and 10-18 years old) from 2000 to 2020. The type of antibiotic prescriptions is indicated with colors (see legend). An overview of the number of cystitis episodes per age category per year (2000-2020) is shown in the table below. y = years old, No. = number of

The type of antibiotic prescriptions varied per age group (Fig. 3). In young children with cystitis (2-4 years and 5-9 years) the number of sulfa-trimethoprim prescriptions decreased from 2000 to 2020 (Additional file 5). In 2020, children below 10 years with cystitis were most often treated with amoxicillin (37.8 95%CI 32.0-43.9%), sulfa-trimethoprim (17.2 95%CI 12.9-22.3%), and amoxicillin clavulanic acid (15.4 95%CI 11.3-20.2%); while 13.9% (95%CI 10.0-18.6%) of prescriptions were nitrofurantoin, as recommended in the Belgian clinical guidelines. In children with cystitis ≥10 years, the number of nitrofurantoin prescriptions increased (*p* < 0.0001) and the use of fluoroquinolone prescriptions decreased (*p* < 0.0001) from 2000 to 2020. In 2020, in this age group 60.8% (95%CI 54.9-66.4%) of prescriptions were nitrofurantoin. Therefore, it appeared that older children with cystitis were more often treated in accordance with the clinical guidelines. In 2020, for pyelonephritis, the most commonly prescribed antibiotics were amoxicillin clavulanic acid (*n* = 19/48), fluoroquinolones (*n* = 11/48) and amoxicillin (9/48).

## Discussion

### Summary

Although there was an increase in the number of UTI episodes in almost all age groups, it is uncertain whether this reflects a true increase of UTI episodes, improved detection or improved data registration. The overall change in incidence rate was low, e.g. the highest increase in cystitis episodes was observed in girls 5-9 years old, where it changed from 19.4 (2000-2002) to 33.4 (2018-2020) episodes per 1000 person-years. The highest increase in pyelonephritis episodes was observed for boys < 2 years, where it changed from 0.9 per 1000 person-years (95%CI 0.1-3.3) (2000-2003) to 3.4 per 1000 person-years (95%CI 2.5-5.0) (2017-2020).

There was an increase in use of laboratory urine tests in children < 10 years and a decrease in number of electronic antibiotic prescriptions in children > 2 years. This may indicate a change in clinical practice: GPs diagnosing UTI now more based on laboratory tests, a decrease in treatment of contaminated samples, an overall decrease in antibiotic use in children [21], an increase in use of compound (non-electronic) prescriptions (such as nitrofurantoin syrup), or GPs referring more children with UTI to secondary care instead of initiating antibiotics.

In children with cystitis < 10 years, the use of broad-spectrum agents was high, and most prescriptions were not in accordance with the Belgian guidelines. This may reflect diagnostic uncertainty in children, for example challenges in differentiating UTI from other acute infections; or cystitis from pyelonephritis.

### Strengths and limitations

A strength of this study was the large sample size and long period of time that data was obtained. Additionally, the population of the Intego database is representative for the Flemish population concerning age and gender [16], which minimizes the risk of selection bias.

A limitation of registry-based studies is that UTI diagnosis depends on the quality of registration by GPs. The incidence rates in this study could have been underestimated because diagnosing UTI is challenging in children and the sensitivity of clinical suspicion is low. In children with a registered diagnosis of cystitis, around 57% had a lab-based urine test available, indicating that UTI diagnosis might have been a working hypothesis based on clinical suspicion or the dipstick test, or that urine collection was often unsuccessful.

Additionally, there was an increase in number of participating practices in 2018, and therefore, differences in registration habits between practices could have influenced our results. In February 2020, the nSARS-CoV2 pandemic started in Belgium. Therefore, the population of children included in 2020 might have consisted of a more severely ill spectrum of children, which could have resulted in an overestimation of the incidence rates of UTI in 2020, and an overestimation of the change in incidence rates of UTIs, from 2000 to 2020.

Only urine test results obtained through the laboratory were available for analysis: meaning that urine dipstick tests and urine microscopy performed at the practice were not included. Therefore, the overall use of urine tests in acutely ill children (boys: 91-224 and girls: 168-383 per 1000 children visiting the general practice) might have been underestimated. However, this seems unlikely as our numbers where higher when compared to data from general practices in the UK (30-40 urine samples per 1000 children) [22].

It remains uncertain why we found a low antibiotic prescribing rate and decrease in use of antibiotic therapy in children with UTI. The overall prescribing rate in children with UTI was comparable to other primary care studies (43-77%) [10, 23–25], and a decrease in antibiotic use in children has also been described previously, based on pharmacy dispensing data in Belgium [21]. One possible explanation could be a high referral rate to secondary care, however in this study, data on other management actions than medication were not available. Two other primary care studies reported referral rates of 8% [10] and 14% [23] in children with UTI, increasing with recurrent UTIs and younger age. These findings suggests that referral to secondary care might partially explain the limited use of antibiotics in our study, especially in young children. Another reason for the low prescribing rate could be a higher use of compound prescriptions, which were not captured in the database. The number of nitrofurantoin prescriptions for cystitis could have been underestimated, because nitrofurantoin syrup is not available as pre-packaged medication in Belgium.

### Comparison with existing literature

The incidence rates of cystitis were comparable with data obtained from general practices in the Netherlands (2001 and 2017), describing incidence rates of 19 and 42 episodes per 1000 person-years [10, 24]. in children < 18 years and 3-5 years old respectively. The variation per age in incidence rates of cystitis was also comparable with other data; showing a peak in cystitis episodes in children between 2 to 10 years, especially in girls [6, 10, 24].

Compared to the incidence rates of cystitis in older registry based-studies in children < 15 year in general practice (1970-1974; 7.7 (girls) and 3.8 (boys) per 1000 person-years) [13], (1979; 1.7 (boys) and 3.1 (girls) per 1000 person-years) [12]; the incidence rates in this study appeared to be higher; indicating that UTI detection might have increased in primary care compared to 50 years ago.

The increase in use of laboratory urine tests found in this study shows similarities with data obtained in the UK, demonstrating an increase in use of urine culture and urine microscopy from 2000 to 2007 followed by a plateau 2008-2015 [26]. In our study, the increase in use was highest starting from 2010. In their study, the use of the urine dipstick test decreased from 2007 to 2015 [26], which could reflect a change in performing urine tests at the practice to performing urine tests at the central laboratory.

The use of broad spectrum antibiotic prescriptions in our study was high, and most prescriptions for cystitis were not in accordance with the Belgian clinical guidelines. These findings are comparable to data obtained in other primary care studies, showing 26-34% inadequate use of antibiotic treatment in children with UTI [10, 24, 25, 27]. Data from Sciensano, the Belgian Scientific Institute of Public Health, show very high resistance of urinary *Escherichia Coli* to amoxicillin (52%) in 2020 [2017: 51%, 2018: 53%, 2019: 50%]; and to amoxicillin clavulanic acid (43%) in 2020 [2017: 16%, 2018: 45%, 2019: 44%], in outpatient children < 5 years old. Resistance to nitrofurantoin, the first choice treatment in Belgium, remains low in children (0%, 2017-2020) [28], but was only used in a minority of children in our study. Decreasing diagnostic uncertainty and improving detection of UTIs in primary care in order to allow targeted therapy is therefore urgently needed.

### Implications for practice and research

Over the last 21 years, there was a slight increase in detection of UTIs in children in primary care, although the overall change in diagnosis was low. There was an increase in use of laboratory urine tests and the use of broad-spectrum agents for lower UTI was high. Therefore, more efforts should be made to improve detection and management of UTI in children. Better adherence to the guidelines is necessary to improve the quality of antibiotic prescribing. Novel point-of-care tests might be useful to improve targeted therapy, by providing the antibiotic susceptibility patterns of urinary pathogens more rapidly than urine culture.

## Supplementary Information

**Additional file 1.** “Table: Number of general practices and population of children per year”. Table including the number of practices per year, participating in the Intego project; the yearly contact group, % boys, % girls, and estimated practice population.

**Additional file 2.** “Table: Incidence rates of cystitis, pyelonephritis and urine testing with 95% confidence intervals per age and gender (2020)”. Table including the estimated incidence rates of cystitis, pyelonephritis and urine testing rate per age and gender.

**Additional file 3.** “Figure: Number of cystitis episodes* per month (2018-2020) *standardized for the variation in yearly population”. Figure showing number of cystitis episodes per month, for the last 3 years available.

**Additional file 4.** “Figure: Incidence rates of laboratory urine tests per cystitis episode per age group from 2000 to 2020”. Figure showing the incidence rates of laboratory urine tests per cystitis episode per age group from 2000 to 2020.

**Additional file 5.** “Table: Results of the autoregressive moving average time series analysis of trends of antibiotic prescriptions and urine testing in children with cystitis from 2000-2020”. Table presenting the results of the autoregressive moving average time series analysis of trends of antibiotic prescriptions and urine testing in children with cystitis from 2000-2020.

## Data Availability

The study protocol is available upon request from the corresponding author. The datasets generated and/or analyzed during the current study are not publicly available due to inclusion of protected health information but can be made available subsequent to further de-identification upon reasonable request from the corresponding author: Jan Y Verbakel.

## Abbreviations

ATC: Anatomical Therapeutic Chemical Classification
GP: General Practitioner
ICPC-2: International Classification of Primary Care, second edition
SAS: Statistical Analysis System
UTI: Urinary tract infection
95%CI: 95% confidence interval

## References

[1] Shaikh N, Haralam MA, Kurs-Lasky M, Hoberman A (2019). Association of Renal Scarring with Number of febrile urinary tract infections in children. JAMA Pediatr.

[2] National Institute for Health and Care Excellence (NICE). Urinary tract infection in under 16s: diagnosis and management (Clinical guideline CG54). London: NICE; 2007. updated Oct 2018; cited 25 Aug 2021

[3] Subcommittee on Urinary Tract Infection (2016). Reaffirmation of AAP clinical practice guideline: the diagnosis and Management of the Initial Urinary Tract Infection in febrile infants and young children 2-24 months of age. Pediatrics..

[4] Boon HA, Van den Bruel A, Struyf T, Gillemot A, Bullens D, Verbakel JY (2021). Clinical features for the diagnosis of pediatric urinary tract infections: systematic review and Meta-analysis. Ann Fam Med.

[5] Stansfeld JM (1966). Clinical observations relating to incidence and aetiology of urinary-tract infections in children. Br Med J.

[6] Winberg J, Andersen HJ, Bergstrom T, Jacobsson B, Larson H, Lincoln K (1974). Epidemiology of symptomatic urinary tract infection in childhood. Acta Paediatr Scand Suppl.

[7] Marild S, Jodal U (1998). Incidence rate of first-time symptomatic urinary tract infection in children under 6 years of age. Acta Paediatr.

[8] Uhari M, Nuutinen M (1988). Epidemiology of symptomatic infections of the urinary tract in children. BMJ..

[9] Pead L, Maskell R (1994). Study of urinary tract infection in children in one health district. BMJ..

[10] Kwok WY, de Kwaadsteniet MC, Harmsen M, van Suijlekom-Smit LW, Schellevis FG, van der Wouden JC (2006). Incidence rates and management of urinary tract infections among children in Dutch general practice: results from a nation-wide registration study. BMC Pediatr.

[11] van de Lisdonk EH, J V. (2000). Kinderen met urineweginfecties: verwijzen of niet?. Huisarts Wet.

[12] Dickinson JA (1979). Incidence and outcome of symptomatic urinary tract infection in children. Br Med J.

[13] Brooks D, Houston IB (1977). Symptomatic urinary infection in childhood: presentation during a four-year study in general practice and significance and outcome at seven years. J R Coll Gen Pract.

[14] Belgische gids voor anti-infectieuze behandeling in de ambulante praktijk (BAPCOC); Belgium: BAPCOC. 2021:33-4. [updated 2021 Jan; cited 17 Sept 2021].

[15] Paschke AA, Zaoutis T, Conway PH, Xie D, Keren R (2010). Previous antimicrobial exposure is associated with drug-resistant urinary tract infections in children. Pediatrics..

[16] Truyers C, Goderis G, Dewitte H, Akker M, Buntinx F (2014). The Intego database: background, methods and basic results of a Flemish general practice-based continuous morbidity registration project. BMC Med Inform Decis Mak.

[17] Benchimol EI, Smeeth L, Guttmann A, Harron K, Moher D, Petersen I (2015). The REporting of studies conducted using observational routinely-collected health data (RECORD) statement. PLoS Med.

[18] Nielen MMJ, Spronk I, Davids R, Korevaar JC, Poos R, Hoeymans N (2019). Estimating morbidity rates based on routine electronic health Records in Primary Care: observational study. JMIR Med Inform.

[19] Enders W (2004). Stationary time-series models. Applied Econometric Time Series.

[20] SAS Institute Inc (2014). SAS/ETS 13.2 User's Guide.

[21] Dillen H, Burvenich R, De Burghgraeve T, Verbakel JY (2022). Using Belgian pharmacy dispensing data to assess antibiotic use for children in ambulatory care. BMC Pediatr.

[22] Cunningham AM, Edwards A, Jones KV, Bourdeaux K, Willock J, Barnes R (2005). Evaluation of a service development to increase detection of urinary tract infections in children. J Eval Clin Pract.

[23] Harmsen M, Wensing M, Braspenning JC, Wolters RJ, van der Wouden JC, Grol RP (2007). Management of children's urinary tract infections in Dutch family practice: a cohort study. BMC Fam Pract.

[24] Dekker ARJ, Verheij TJM, van der Velden AW (2017). Antibiotic management of children with infectious diseases in Dutch primary care. Fam Pract.

[25] Copp HL, Shapiro DJ, Hersh AL (2011). National ambulatory antibiotic prescribing patterns for pediatric urinary tract infection, 1998-2007. Pediatrics..

[26] O'Sullivan JW, Stevens S, Hobbs FDR, Salisbury C, Little P, Goldacre B (2018). Temporal trends in use of tests in UK primary care, 2000-15: retrospective analysis of 250 million tests. BMJ..

[27] Al-Sayyed B, Le J, Al-Tabbaa MM, Barnacle B, Ren J, Aiyer M (2017). Uncomplicated urinary tract infection (UTI) in ambulatory primary care pediatrics. Are we using antibiotics appropriately?. Open Forum Infect Dis Ther.

[28] Mertens K, Catteau L (2020). European antimicrobial resistance surveillance for Belgium (EARS-BE) 2020 , Report No D/2021/14.440/90.

