# Supplementary material for: Incidence rates and trends of childhood urinary tract infections and antibiotic prescribing: registry-based study in general practices (2000 to 2020)
Source: BMC Prim Care. 2022 Jul 20;23:177. doi: 10.1186/s12875-022-01784-x (PMC9301837; doi:10.1186/s12875-022-01784-x)
Supplement: Supplementary file 1 — Additional file 1. “Table: Number of general practices and population of children per year”. Table including the number of practices per year, participating in the Intego project; the yearly contact group, % boys, % girls, and estimated practice population. [file 12875_2022_1784_MOESM1_ESM.pdf]

Additional file 1: Number of general practices and population of children per year

Year	Number of practices	Yearly contact group	**Boys** (%)	**Girls** (%)	Estimated practice population
2000	65	16330	8407 (51.5%)	7923 (48.5%)	23749
2001	63	15563	8008 (51.5%)	7555 (48.5%)	22452
2002	69	19052	9795 (51.4%)	9257 (48.6%)	27451
2003	72	22805	11855 (42.5%)	10950 (57.5%)	32664
2004	74	21088	10964 (52.0%)	10124 (48.0%)	30351
2005	74	22625	11737 (51.9%)	10888 (48.1%)	32685
2006	74	23571	12239 (51.9%)	11332 (48.1%)	34191
2007	72	23537	12124 (51.5%)	11413 (48.5%)	34167
2008	71	23800	12186 (51.2%)	11614 (48.8%)	34361
2009	69	25418	13109 (51.6%)	12309 (48.4%)	36557
2010	68	24921	12866 (51.6%)	12055 (48.4%)	38075
2011	66	26914	13879 (51.6%)	13035 (48.4%)	40221
2012	61	22561	11697 (51.8%)	10864 (48.2%)	33597
2013	55	23850	12243 (51.3%)	11607 (48.7%)	35314
2014	49	23634	12164 (51.5%)	11470 (48.5%)	35602
2015	49	22777	11623 (51.0%)	11154 (49.0%)	33701
2016	40	21749	11194 (51.5%)	10555 (48.5%)	31370
2017	45	21432	10870 (50.7%)	10562 (49.3%)	28793
2018	105	54452	27704 (50.9%)	26748 (49.1%)	67722
2019	104	55959	28548 (51.0%)	27411 (49.0%)	69918
2020	104	56651	29035 (51.3%)	27616 (48.7%)	23749

Table showing the yearly number of practices, yearly contact group (all children up to 18 years of age that visited the general practice at least once in that year), number of boys/girls included and the estimated practice population. The practice population is estimated by applying a correction factor to the yearly contact group after stratification for age, gender and geographical region, based on data provided annually by the Belgian inter-mutualistic agency.
