# Supplementary material for: Incidence rates and trends of childhood urinary tract infections and antibiotic prescribing: registry-based study in general practices (2000 to 2020)
Source: BMC Prim Care. 2022 Jul 20;23:177. doi: 10.1186/s12875-022-01784-x (PMC9301837; doi:10.1186/s12875-022-01784-x)
Supplement: Supplementary file 2 — Additional file 2. “Table: Incidence rates of cystitis, pyelonephritis and urine testing with 95% confidence intervals per age and gender (2020)”. Table including the estimated incidence rates of cystitis, pyelonephritis and urine testing rate per age and gender. [file 12875_2022_1784_MOESM2_ESM.pdf]

Additional file 2: Incidence rates of cystitis, pyelonephritis and urine testing with 95% confidence intervals per age and gender (2020)

**Infection**	**Age group (Years)**	**Gender**	**Incidence** (/1000 person-years)	**lower 95%CI**	**upper 95%CI**
cystitis (ICPC code U71)	0-1	boys	4.744392	2.593803	7.960284
		girls	8.479613	5.433048	12.61699
	2-4	boys	5.230675	3.351392	7.78283
		girls	40.27462	34.54415	46.68412
	5-9	boys	3.913372	2.676744	5.524514
		girls	32.79605	28.81763	37.17021
	10-18	boys	2.555856	1.770005	3.571553
		girls	24.16318	21.54794	27.00832
pyelonephritis (ICPC code U70)	0-1	boys	5.422727	3.099557	8.806168
		girls	4.943761	2.7028	8.294791
	2-4	boys	0.435569	0.052749	1.573426
		girls	5.462842	3.500146	8.128276
	5-9	boys	0.488915	0.133213	1.251816
		girls	2.531617	1.524199	3.953433
	10-18	boys	0.075144	0.001902	0.418674
		girls	1.398141	0.828627	2.209665
Urine testing*	0-1	boys	142.66604	129.33100	157.00276
		girls	145.47911	131.73639	160.26587
	2-4	boys	203.44813	190.56053	216.97798
		girls	352.67148	335.21934	370.79653
	5-9	boys	110.34035	103.24452	117.79542
		girls	243.05479	231.97917	254.52258
	10-18	boys	80.50027	75.74414	85.47682
		girls	163.00709	156.08624	170.15579

*Urine testing was defined as ≥1 of the following tests results obtained from the laboratory: urine dipstick test, urine microscopy, urine culture and/or antibiogram

95%CI = 95% confidence intervals, ICPC= International Classification of Primary Care
