# Supplementary material for: Incidence rates and trends of childhood urinary tract infections and antibiotic prescribing: registry-based study in general practices (2000 to 2020)
Source: BMC Prim Care. 2022 Jul 20;23:177. doi: 10.1186/s12875-022-01784-x (PMC9301837; doi:10.1186/s12875-022-01784-x)
Supplement: Supplementary file 3 — Additional file 3. “Figure: Number of cystitis episodes* per month (2018-2020) *standardized for the variation in yearly population”. Figure showing number of cystitis episodes per month, for the last 3 years available. [file 12875_2022_1784_MOESM3_ESM.pdf]

Additional file 3: Number of cystitis episodes* per month (2018-2020)

***standardized for the variation in yearly population**

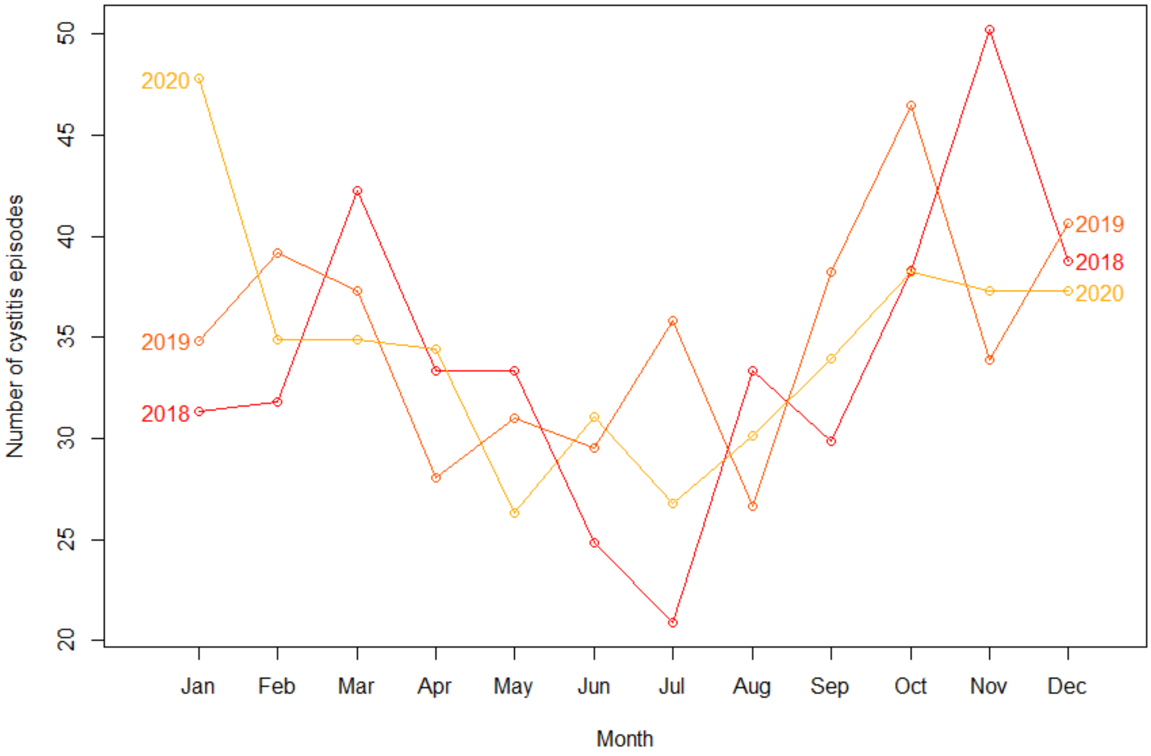

Number of cystitis episodes per month (2018-2020), based on number of episodes standardized for the variation in yearly population (plot made using package ‘Forecast’ in R).
