# Supplementary material for: Incidence rates and trends of childhood urinary tract infections and antibiotic prescribing: registry-based study in general practices (2000 to 2020)
Source: BMC Prim Care. 2022 Jul 20;23:177. doi: 10.1186/s12875-022-01784-x (PMC9301837; doi:10.1186/s12875-022-01784-x)
Supplement: Supplementary file 4 — Additional file 4. “Figure: Incidence rates of laboratory urine tests per cystitis episode per age group from 2000 to 2020”. Figure showing the incidence rates of laboratory urine tests per cystitis episode per age group from 2000 to 2020. [file 12875_2022_1784_MOESM4_ESM.pdf]

Additional file 4: Incidence rates of laboratory urine tests per cystitis episode per age group from 2000 to 2020

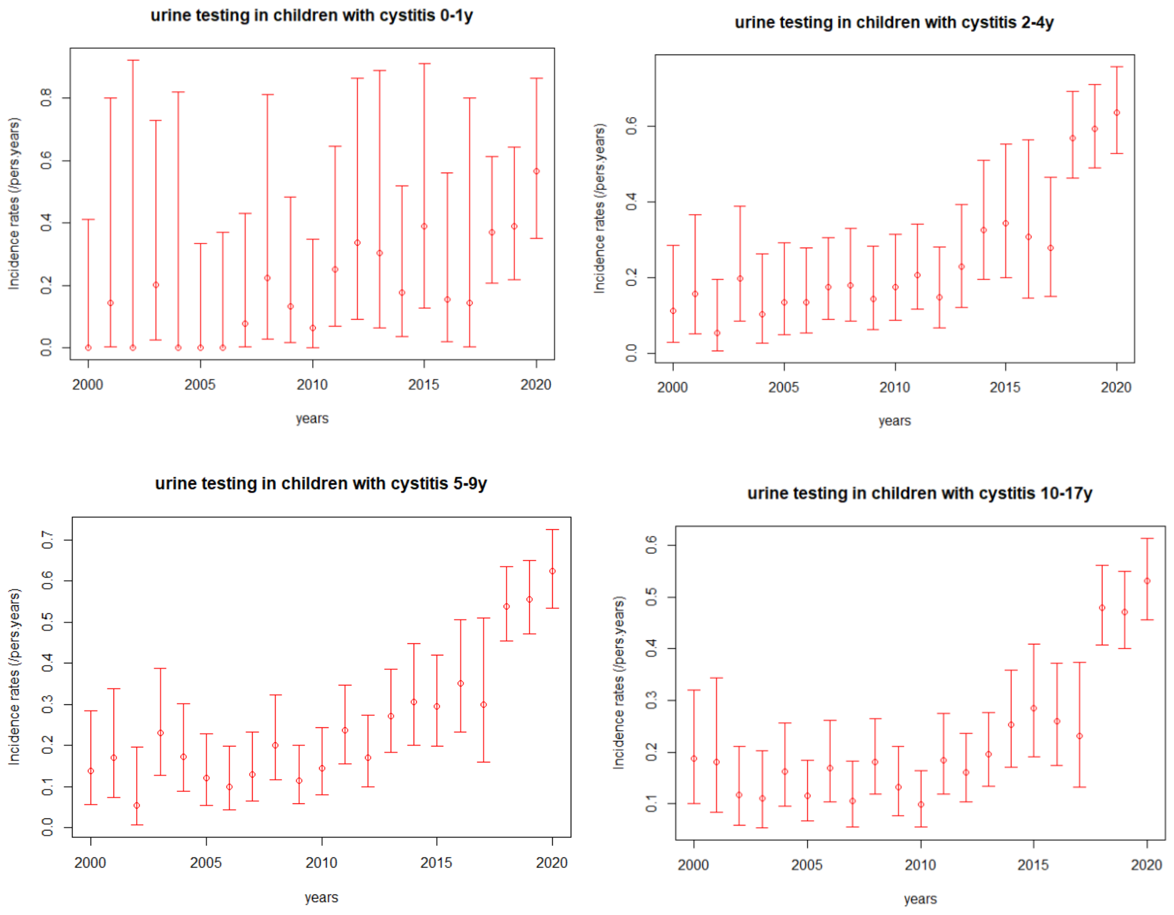

Most recent year 2020*

**Age** (years)	**Incidence laboratory urine test per cystitis episode** (/1 person-years) (95% CI)
0-1	0.5646 (0.349495861-0.8630500)
2-4	0.6351 (0.528246048-0.7572291)
5-9	0.6247 (0.534088422-0.7263418)
10-17	0.5310 (0.456011458-0.6146799)

95%CI= 95% confidence intervals
