# Supplementary material for: Incidence rates and trends of childhood urinary tract infections and antibiotic prescribing: registry-based study in general practices (2000 to 2020)
Source: BMC Prim Care. 2022 Jul 20;23:177. doi: 10.1186/s12875-022-01784-x (PMC9301837; doi:10.1186/s12875-022-01784-x)
Supplement: Supplementary file 5 — Additional file 5. “Table: Results of the autoregressive moving average time series analysis of trends of antibiotic prescriptions and urine testing in children with cystitis from 2000-2020”. Table presenting the results of the autoregressive moving average time series analysis of trends of antibiotic prescriptions and urine testing in children with cystitis from 2000-2020. [file 12875_2022_1784_MOESM5_ESM.pdf]

Additional file 5: Results of the autoregressive moving average time series analysis of trends of antibiotic prescriptions and urine testing in children with cystitis from 2000-2020

Variable	Age group (Years)	Annual change in prescription rate[1] (/person-year (95%CI))	P-value
total number of antibiotic prescriptions within 14 days before to 14 days after diagnosis	0-1	-0.0123 (-0.0319 to 0.0073)	0.2174
	2-4	**-0.0194 (-0.0293 to -0.0094)**	**0.0010**
	5-9	**-0.0215 (-0.0281 to -0.01484)**	**<0.0001**
	10-18	**-0.0060 (-0.0114 to -0.0006)**	**0.0359**
amoxicillin	2-4	+0.0055 (-0.0026 to 0.0136)	0.1825
sulfa-trimethoprim	2-4	**-0.0156 (-0.0241 to -0.0072)**	**0.0016**
amoxicillin	5-9	+0.0032 (-0.005 to 0.0117)	0.4628
sulfa-trimethoprim	5-9	**-0.0185 (-0.0229 to -0.0141)**	**<0.0001**
nitrofurantoin	10-18	**+0.0213 (0.0197 to 0.0230)**	**<0.0001**
fluoroquinolones	10-18	**-0.0184 (-0.0211 to -0.0211)**	**<0.0001**
Laboratory tests performed within 14 days before to 14 days after UTI diagnosis	0-1	**+0.0194 (0.0115 to 0.0274)**	**<0.0001**
	2-4	**+0.0236 (0.0080 to 0.0393)**	**0.0069**
	5-9	**+0.0223 (0.0045 to 0.0401)**	**0.0208**
	10-18	+ 0.0163 (-0.0019 to 0.0346)	0.0852

[1]Mean annual change in incidence rate as estimated by the model; Statistically significant changes are indicated in **bold** (p<0.05)

95%CI = 95% confidence intervals
